# From conceptual understanding to interdisciplinary innovation: the mediating mechanism of IESR among high school students

**DOI:** 10.3389/fpsyg.2026.1739020

**Published:** 2026-05-20

**Authors:** Jiyuan Xu, Yurui Mou, Lei Yuan

**Affiliations:** 1 Faculty of Education, Guangxi Normal University, Guilin, China; 2 Research and Development Center for Educational Informatization, Guangxi Normal University, Guilin, China; 3 Ziyang College of Dental Technology, Ziyang, China; 4 Key Laboratory of Educational Blockchain and Intelligent Technology, Ministry of Education, Guangxi Normal University, Guilin, China

**Keywords:** abstract thinking, conceptual understanding, interdisciplinary integration, mediating effect, practical innovation, scientific inquiry, strategic regulation

## Abstract

Cultivating adolescents' interdisciplinary innovation capability has become a central concern in secondary education, yet the psychological mechanism through which abstract conceptual resources are transformed into authentic innovative practice remains insufficiently understood. Drawing on theories of self-regulated learning, knowledge construction, and learning transfer, this study examined the relationships among high school students' abstract thinking and conceptual understanding (ATCU), inquiry execution and strategic regulation (IESR), and interdisciplinary integration and practical innovation (IIPI), with particular attention to the mediating role of IESR. A total of 2,466 students in grades 10 to 12 from G Province, China, completed a 27-item self-report instrument adapted from the Watson-Glaser Critical Thinking Appraisal, the Torrance Tests of Creative Thinking, and the PISA 2015 Science Literacy Assessment Framework. The instrument demonstrated strong reliability (Cronbach's alpha = 0.977) and good construct validity. Structural equation modeling and bootstrap mediation analyses based on 5,000 resamples yielded four main findings: (1) ATCU significantly predicted IIPI (β = 0.220, *p* < 0.001); (2) ATCU significantly predicted IESR (β = 0.769, *p* < 0.001); (3) IESR significantly predicted IIPI (β = 0.704, *p* < 0.001); and (4) IESR partially mediated the relationship between ATCU and IIPI (total effect = 0.756; direct effect = 0.220, 29.10%; indirect effect = 0.536, 70.90%; 95% CI [0.509, 0.563]). The model explained 74.9% of the variance in IIPI. These findings suggest that abstract conceptual understanding contributes to interdisciplinary innovation primarily through inquiry execution and metacognitive regulation, forming an “understanding-inquiry-reflection” cycle that is central to the development of adolescents' scientific literacy. This study extends metacognitive theory into the domain of interdisciplinary innovation and offers evidence-based implications for competency-based STEM curriculum design, instructional intervention, and assessment reform.

## 1 Introduction

Since the beginning of the 21st century, the accelerating pace of scientific and technological innovation has profoundly reshaped the capability structure demanded by the global knowledge economy (OECD, 2019; World Economic Forum, 2020). In response, education systems worldwide have increasingly shifted their focus from the transmission of fragmented disciplinary knowledge to the cultivation of interdisciplinary innovation capabilities. STEM is now widely regarded as an integrated meta-disciplinary framework that emphasizes innovation and the application of knowledge to the design of solutions for complex contextualized problems, while also fostering problem-solving and critical thinking skills (National Academies of Sciences, 2024). Consequently, integrated STEM education and project-based learning have emerged as mainstream instructional approaches for strengthening students' capacity to solve real-world problems (Bybee, 2013; English, 2016; Hwang et al., 2024). This pedagogical shift reflects not only a curricular preference but also a strategic response to the United Nations Sustainable Development Goals, especially SDG 4 on quality education and SDG 9 on industry, innovation, and infrastructure, both of which emphasize the development of higher-order competencies needed to address global challenges (UNESCO, 2024). At the same time, troubling evidence from PISA indicates that across OECD member and partner countries, mean mathematics performance declined by a record 15 points and reading performance fell by 10 points between 2018 and 2022 (OECD, 2023). These findings underscore the urgent need to rethink secondary education so that it prioritizes authentic competency development rather than rote acquisition of knowledge.

This challenge is especially pressing at the high school level, a developmental period marked by the maturation of higher-order thinking and by critical decisions related to academic and career trajectories (Inhelder and Piaget, 1958; Kuhn, 2009; Poll and Petru, 2023). Although contemporary adolescents often demonstrate substantial disciplinary knowledge acquisition (Mullis et al., 2020), they frequently struggle when confronted with novel interdisciplinary problems that require knowledge transfer and adaptive application (Barnett and Ceci, 2002; Kim and Joo, 2023). Abstract conceptual understanding alone is not sufficient to generate innovative solutions to complex real-world issues such as climate change and public health crises (Honey et al., 2014). This persistent knowledge-action gap not only limits students' readiness for higher education and their adaptability in future careers, but may also weaken national capacity for research and innovation (Marginson et al., 2013). Although a large body of research has established the importance of cognitive abilities for academic achievement, three critical gaps remain. First, most existing studies have focused on the direct effects of metacognition on academic performance, while paying insufficient attention to its mediating role in transforming higher-order cognitive resources into innovative outcomes (Schukajlow et al., 2023). Second, research on interdisciplinary innovation has largely concentrated on instructional interventions and curriculum design (Kelley and Knowles, 2016), whereas the underlying psychological mechanisms, particularly the ways in which learners internally coordinate abstract concepts, inquiry execution, and strategic regulation, remain insufficiently examined. Third, large-scale empirical evidence specific to the high school developmental stage is still limited, leaving the pathway from understanding to innovation during adolescence inadequately explained.

To address these gaps, the present study proposes and empirically tests a theoretical model in which Inquiry Execution and Strategic Regulation (IESR) mediates the relationship between Abstract Thinking and Conceptual Understanding (ATCU) and Interdisciplinary Integration and Practical Innovation (IIPI) among 2,466 high school students. Drawing on self-regulated learning theory (Zimmerman, 2002), knowledge construction theory (Bereiter and Scardamalia, 2014), and learning transfer theory (Barnett and Ceci, 2002), this study aims to clarify the psychological pathway through which abstract conceptual resources are transformed into authentic innovative practice. From a theoretical perspective, the study contributes to educational psychology by articulating an “understanding, inquiry, and reflection” cycle that explains how declarative knowledge develops into procedural competence and ultimately into innovative output. In doing so, it extends metacognitive theory to the context of interdisciplinary innovation. From a practical perspective, the findings are expected to provide evidence-based implications for curriculum design, instructional intervention, and assessment reform, particularly in support of competency-based STEM education aligned with SDG 4 and SDG 9. Identifying the psychological mechanisms that connect abstract thinking with actual interdisciplinary innovation outcomes is therefore important not only as a theoretical concern in educational psychology but also as a practical necessity for cultivating the innovators required by an increasingly complex global society.

## 2 Literature review

### 2.1 Abstract thinking and conceptual understanding

Abstract Thinking and Conceptual Understanding (ATCU) refers to students' higher-order cognitive ability to transform concrete phenomena into abstract symbolic systems, identify logical relationships among concepts, and understand the boundary conditions of theoretical applicability (Inhelder and Piaget, 1958). The core of this ability lies in transcending surface information to grasp the essential structure and underlying principles of disciplinary knowledge. It comprises three key components: conceptual abstraction, the ability to extract universal features from concrete instances and form abstract concepts; relational reasoning, the ability to identify and manipulate logical relationships among concepts, including causal, analogical, and hierarchical relationships (Krawczyk, 2018) and theoretical framework understanding, the ability to grasp the overall structure of disciplinary knowledge systems and understand the scope and boundary conditions of theoretical applicability.

Existing research demonstrates that ATCU is an important predictor of academic achievement. (Adey and Shayer 1994) cognitive acceleration intervention research confirmed that enhancing students' abstract reasoning abilities can significantly promote academic performance across multiple disciplines. Recent studies further reveal that deep conceptual understanding is the foundation of effective problem-solving: the quality of conceptual understanding significantly predicts innovative performance in STEM fields (Beymer et al., 2023) high conceptual understanders focus more on deep structure rather than surface features when problem-solving. However, while the association between ATCU and academic performance has been well established, how it transforms into interdisciplinary innovation capability in authentic contexts remains an unsolved mystery. (Barnett and Ceci 2002) pointed out that the success rate of knowledge transfer decreases sharply with increasing contextual differences; merely possessing abstract conceptual understanding is insufficient to guarantee flexible knowledge application (Dunlosky et al., 2024). This “transfer dilemma” prompts researchers to explore the psychological mechanisms connecting abstract thinking with actual innovation.

### 2.2 Inquiry execution and strategic regulation

Inquiry Execution and Strategic Regulation (IESR) refers to students' metacognitive ability to plan, monitor, evaluate, and adjust their cognitive activities during inquiry-based learning processes (Zimmerman, 2002). This ability is essentially the contextualization of metacognitive theory in scientific inquiry situations. (Flavell 1979) defined metacognition as “cognition about cognition,” encompassing metacognitive knowledge and metacognitive regulation. Specifically, IESR encompasses four core dimensions (Schraw and Dennison, 1994; Van der Stel and Veenman, 2010): inquiry planning, the prospective ability to set inquiry goals, analyze task requirements, select appropriate strategies, and allocate cognitive resources; process monitoring, the real-time regulation ability to continuously track progress, detect comprehension deviations, and identify strategy effectiveness during inquiry execution; strategic adjustment, the adaptive ability to flexibly modify inquiry methods, revise hypotheses, or seek alternative pathways based on monitoring feedback; and reflective evaluation, the retrospective ability to review the overall process after inquiry completion, summarize lessons learned, and extract transferable principles.

Metacognitive ability is widely regarded as the core mechanism of learning efficacy. Metacognitive training has significant positive effects on academic achievement (Dignath and Büttner, 2008). Recent research further confirms that metacognitive scaffolding-based scientific inquiry instruction can significantly enhance students' problem-solving abilities and deep learning performance (Azevedo, 2018) students with high metacognitive levels demonstrate stronger strategic flexibility and error detection capabilities in scientific inquiry (van der Stel and Veenman, 2014) metacognitive monitoring plays a key mediating role in complex problem-solving, particularly in interdisciplinary contexts requiring knowledge integration (Schukajlow et al., 2023). However, existing research primarily focuses on the direct impact of metacognition on academic performance, with less exploration of its mediating role in higher-order ability transformation processes. Particularly in interdisciplinary innovation contexts, where students face complex challenges such as knowledge integration and coordination of multiple perspectives, how IESR facilitates the transformation of abstract thinking into innovative practice still lacks systematic empirical examination.

### 2.3 Interdisciplinary integration and practical innovation

Interdisciplinary Integration and Practical Innovation (IIPI) refers to students' comprehensive ability to integrate multidisciplinary knowledge, perspectives, and methods to solve complex real-world problems and generate novel solutions (Klein, 2005; Swiecki et al., 2022). This ability transcends single disciplinary boundaries, requiring students to identify the complementarity of knowledge from different fields, establish interdisciplinary connections, and apply integrated knowledge to actual innovative practice. (Boix Mansilla 2005) pointed out that true interdisciplinary understanding must meet three criteria: purposefulness, disciplinary grounding, and integration. At the operational level, IIPI encompasses three progressive stages (Repko and Szostak, 2020): knowledge identification and connection, identifying the multidisciplinary knowledge required for problems, understanding the unique contributions of each discipline, and establishing bridges between concepts; perspective integration and conflict resolution, integrating different disciplinary perspectives, reconciling assumption conflicts or methodological differences between disciplines, and forming a unified framework; and innovative application and solution generation, applying integrated knowledge to novel contexts, designing practical solutions, and evaluating their feasibility and innovativeness.

Recent research shows that interdisciplinary problem-solving is significantly positively correlated with creative thinking, as authentic innovation often occurs at disciplinary intersections (Kelley and Knowles, 2016) conceptual integration ability in scientific innovation teams is a key predictor of breakthrough discoveries (Nersessian and Patton, 2023) interdisciplinary project-based learning in STEM fields significantly enhances students' innovative thinking and problem-solving abilities (Kwon and Lee, 2025). However, many students exhibit a “knowledge island” phenomenon in interdisciplinary tasks-despite mastering disciplinary knowledge, they cannot effectively integrate it (Spelt et al., 2009) even in contexts explicitly requiring interdisciplinary thinking, students still tend to adopt single-disciplinary perspectives (Darbellay, 2015). These findings suggest that IIPI does not automatically emerge from disciplinary knowledge accumulation but requires specific cognitive and metacognitive mechanisms to support cross-boundary knowledge transfer and creative recombination (Darbellay, 2022).

### 2.4 Theoretical integration and conceptual model construction

To understand the intrinsic connections among ATCU, IESR, and IIPI, this study integrates self-regulated learning theory, knowledge construction theory, and learning transfer theory to construct a theoretical model of mediating mechanisms.

Self-regulated learning theory (Zimmerman, 2000; Panadero, 2017) posits that effective learning is a cyclical self-regulatory process: learners set goals and activate relevant knowledge, execute strategies and monitor progress, reflect on results and adjust methods. Within this framework, metacognitive strategies act as the “commander of cognition,” regulating the operation of other cognitive processes (Flavell, 1979). Specifically, ATCU constitutes students' cognitive resource foundation, providing the knowledge structure needed for problem-solving; IIPI represents explicit learning outcomes, embodying the ultimate manifestation of knowledge transfer and application. The unique value of self-regulated learning theory lies in revealing the intermediate transformation mechanism: students do not automatically transform abstract concepts into innovative practice but rather need to actively activate, organize, and restructure existing knowledge to adapt to new contexts through metacognitive regulation-including inquiry planning, execution monitoring, strategic flexible adjustment, and reflective evaluation.

Knowledge construction theory provides a complementary perspective for understanding this transformation process. (Bereiter and Scardamalia 2014) emphasized that deep learning is a process where learners actively construct meaning, identify contradictions, and reorganize conceptual networks; innovative learning requires learners to view knowledge as an improvable object, deepening cognition through continuously raising questions, testing ideas, and refining understanding.

Learning transfer theory further reveals the critical conditions for knowledge application. Barnett and Ceci's (2002) transfer taxonomy framework indicates that far transfer requires learners to abstract underlying principles, identify structural similarities, and flexibly adapt strategies. Recent research finds that metacognitive awareness significantly predicts the success rate of learning transfer, particularly in interdisciplinary problem-solving contexts (Phung et al., 2025) high-metacognitive learners are better at identifying deep structural similarities and activating relevant knowledge in new contexts (Wiedbusch et al., 2025).

Based on the above theoretical integration, this study proposes a theoretical model in which IESR plays a key mediating role in the relationship between ATCU and IIPI. This model encompasses three mechanistic pathways: First, the cognitive activation pathway-high levels of ATCU enable students to establish more refined conceptual networks, which provide richer “raw materials” for metacognitive monitoring, allowing students to more accurately assess “what I understand” and “what I need,” thereby formulating more effective inquiry plans. Second, the strategic adaptation pathway-IESR enables students to continuously monitor inquiry progress, identify obstacles in knowledge integration, and flexibly adjust strategies to transform abstract disciplinary knowledge into contextualized problem-solving actions. Third, the resilience maintenance pathway-the self-reflection and emotional regulation components of metacognition enable students to extract experience from failures, maintain motivation, and persist in exploration, ultimately achieving innovative breakthroughs.

## 3 Research objectives

**Figure 1:**
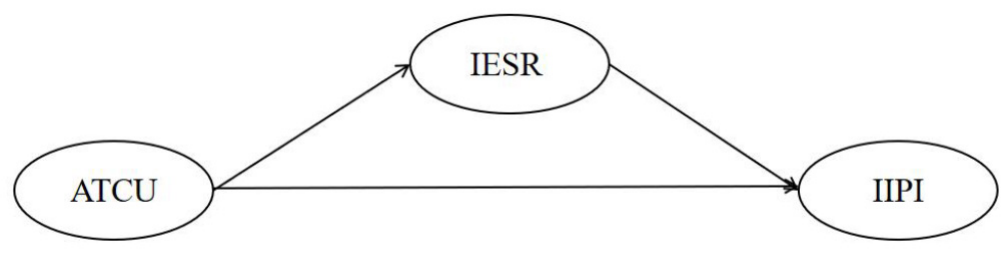
Hypothesized theoretical model.

This study proposes the following research hypotheses and constructs the theoretical model shown in Figure 1:

H1: ATCU has a significant positive predictive effect on IIPI.

H2: ATCU has a significant positive predictive effect on IESR.

H3: IESR has a significant positive predictive effect on IIPI.

H4: IESR plays a partial mediating role in the influence of ATCU on IIPI.

## 4 Methods

### 4.1 Participants and context

Participants were 2,466 high school students from grades 10 to 12 in G Province. According to the sample size requirements for structural equation modeling analysis, the sample size should be at least 10–20 times the number of measurement items (Kline, 2016). The sample size of this study (*N* = 2,466) far exceeds the requirement (28 items × 20 = 560). In terms of sample composition, regarding gender, there were 1,083 male students (43.9%) and 1,383 female students (56.1%). Regarding grade level, there were 1,090 tenth-grade students (44.2%), 651 eleventh-grade students (26.4%), and 725 twelfth-grade students (29.4%). Regarding school type, there were 1,427 students from urban high schools (57.9%), 858 students from county high schools (34.8%), and 181 students from rural high schools (7.3%).

### 4.2 Measures

This study adapted instruments based on the Watson-Glaser Critical Thinking Appraisal (Watson and Glaser, 1980), the Torrance Tests of Creative Thinking (TTCT) (Torrance, 1974), and the PISA 2015 Science Literacy Assessment Framework. The formal scale includes three dimensions with a total of 27 items, using a 5-point Likert scale, where higher scores indicate stronger ability in that dimension. Reliability and validity test results showed: the Cronbach's α coefficient for the overall scale was 0.977, indicating extremely high internal consistency reliability; the KMO value was 0.985, and Bartlett's test of sphericity was significant (χ^2^ = 65056.671, df = 378, *p* < 0.001), indicating that the data were highly suitable for factor analysis; three principal components were extracted with a cumulative variance explained reaching a satisfactory level, indicating good construct validity of the scale.

Abstract Thinking and Conceptual Understanding (ATCU, 5 items): This dimension was constructed based on Piaget's cognitive development theory and the revised Bloom's taxonomy of cognitive objectives by (Anderson et al. 2001), adapted from the reasoning and deduction dimensions of the Watson-Glaser Critical Thinking Appraisal. Abstract thinking is the cognitive foundation of innovation, and high school students are in the formal operational stage, possessing the ability to transform from concrete to abstract (Inhelder and Piaget, 1958). This dimension primarily measures students' ability to transform concrete phenomena into abstract concepts, ability to understand the applicable boundaries of concepts, ability to discover patterns and conduct inductive reasoning, and ability to use thinking tools to analyze complex problems. The Cronbach's α coefficient for this dimension was 0.895, indicating good internal consistency.

Inquiry Execution and Strategic Regulation (IESR, 12 items): This dimension was constructed based on Zimmerman's (2000) self-regulated learning theory and the metacognitive theoretical framework of (Schraw and Dennison 1994), adapted from the scientific inquiry dimension in the PISA 2015 Science Literacy Assessment Framework and the Metacognitive Awareness Inventory (MAI). Metacognitive ability is considered a higher-order ability for learners to monitor and regulate their own cognitive processes, which is crucial for innovative problem-solving (Flavell, 1979). This dimension primarily measures students' abilities in experimental design and adjustment, team collaboration and division of labor, learning strategy regulation, flexibility in problem-solving, research planning and execution, and self-reflection and improvement during scientific inquiry processes. The Cronbach's α coefficient for this dimension was 0.963, indicating extremely high internal consistency.

Interdisciplinary Integration and Practical Innovation (IIPI, 10 items): This dimension was constructed based on STEAM education concepts (Yakman, 2007; Amabile, 1996) componential model of creativity, adapted from the fluency, flexibility, and originality dimensions of the Torrance Tests of Creative Thinking (TTCT), combined with interdisciplinary integration ability assessment tools. Interdisciplinary integration is regarded as an important component of 21st-century core competencies and a key ability for solving complex real-world problems (OECD, 2018). This dimension primarily measures students' ability to analyze problems using multidisciplinary knowledge, ability to integrate and apply knowledge from different disciplines, ability to explain real-world phenomena using disciplinary knowledge, and ability to use information technology to integrate disciplinary learning. The Cronbach's α coefficient for this dimension was 0.953, indicating extremely high internal consistency.

### 4.3 Data analysis

This study used SPSS 26.0 and AMOS 24.0 for data analysis. To test for common method bias, Harman's single-factor test was employed by conducting an unrotated exploratory factor analysis on the 27 items. Results showed that the variance explained by the first factor was 62.268%, which did not exceed the critical value of 70%, indicating that common method bias was within an acceptable range.

Pearson correlation analysis was used to examine the correlational relationships among ATCU, IESR, and IIPI. AMOS 24.0 was used to conduct confirmatory factor analysis (CFA) and structural equation modeling to verify the fit of the three-factor chain mediation model of high school students' innovation literacy. All fit indices of the three-factor model reached ideal standards: χ^2^/df = 2.847 < 3, CFI = 0.982 > 0.95, TLI = 0.980 > 0.95, RMSEA = 0.027 < 0.05, SRMR = 0.022 < 0.08, indicating that the three dimensions of ATCU, IESR, and IIPI had good discriminant validity and the measurement model fit well.

Following the nonparametric percentile bootstrap method proposed by (Hayes 2018), the SPSS macro PROCESS 4.1 Model 4 was used to test the mediating effect of IESR between ATCU and IIPI. The bootstrap sampling number was set to 5,000 times, with a confidence interval (CI) set at 95%.

## 5 Results

### 5.1 Descriptive statistics and correlations

To understand the basic characteristics and interrelationships of the research variables, descriptive statistics and correlation analysis were conducted for ATCU, IESR, and IIPI. Results showed (see Table 1) that the mean scores of the three variables were all at moderate to upper-moderate levels (M = 2.931–3.139), with IESR having the highest score (M = 3.139) and IIPI having a slightly lower score (M = 2.931). Correlation analysis indicated that all three variables were significantly positively correlated with each other (*r* = 0.746–0.854, *p* < 0.01), with the correlation coefficient between IESR and IIPI being the highest (*r* = 0.854), providing a statistical prerequisite for subsequent mediation effect testing.

**Table 1:** Descriptive statistics and correlation coefficient matrix for each variable.

Variable	M	SD	1	2	3
ATCU	2.996	0.900	1		
IESR	3.139	0.891	0.769^**^	1	
IIPI	2.931	0.913	0.746^**^	0.854^**^	1

*N* = 2,466; ATCU, Abstract Thinking and Conceptual Understanding; IESR, Inquiry Execution and Strategic Regulation; IIPI, Interdisciplinary Integration and Practical Innovation; ^**^p < 0.01 (two-tailed test).

### 5.2 Direct effects of ATCU and IESR on IIPI

Multiple regression analysis was used to examine the predictive effects of ATCU and IESR on IIPI. Results showed (see Table 2) that the two predictor variables jointly explained 74.9% of the total variance in IIPI (*R*^2^ = 0.749, F = 3679.751, *p* < 0.001). ATCU had a significant positive predictive effect on IIPI (β = 0.220, *p* < 0.001), and IESR also had a significant positive predictive effect on IIPI (β = 0.704, *p* < 0.001). From the standardized coefficients, the predictive effect of IESR was stronger than that of ATCU, indicating that IESR is a more important predictor of IIPI.

**Table 2:** Multiple regression analysis results of ATCU and IESR on IIPI.

Predictor variable	B	SE	β	*t*	*p*	95% CI	VIF
Constant	0.062	0.035	–	1.782	< 0.075	[−0.006, 0.131]	–
ATCU	0.220	0.016	0.22	13.749	< 0.001	[0.189, 0.252]	2.445
IESR	0.704	0.016	0.704	43.525	< 0.001	[0.672, 0.736]	2.445

*N* = 2,466; *R*^2^ = 0.749, Adjusted *R*^2^ = 0.749, F(2, 2463) = 3679.751^***^; B, unstandardized regression coefficient; β, standardized regression coefficient; VIF, variance inflation factor; ATCU, Abstract Thinking and Conceptual Understanding; IESR, Inquiry Execution and Strategic Regulation; IIPI, Interdisciplinary Integration and Practical Innovation; ^***^p < 0.001.

### 5.3 Mediating role of IESR

**Figure 2:**
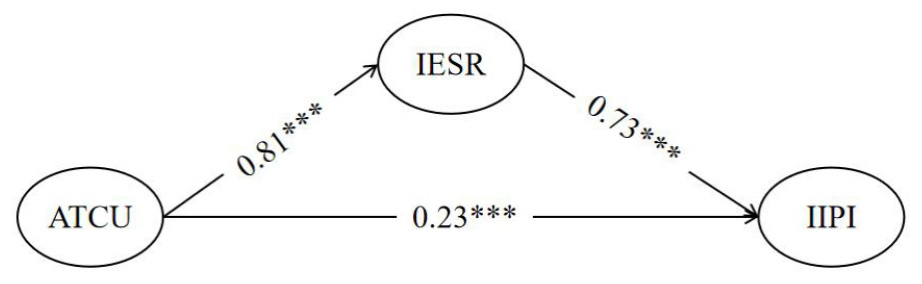
Mediation effect model of IESR in the relationship between ATCU and IIPI. All path coefficients are significant (*p* < 0.001); values are standardized coefficients.

The PROCESS macro (Model 4) and bootstrap method (5,000 repeated samples) were used to test the mediating role of IESR (see Table 3), and the mediation model was successfully constructed in AMOS (see Figure 2). Results showed that ATCU had a significant positive predictive effect on IESR (β = 0.769, *p* < 0.001), IESR had a significant positive predictive effect on IIPI (β = 0.704, *p* < 0.001), and the direct effect of ATCU on IIPI remained significant after controlling for IESR (β = 0.220, *p* < 0.001). Decomposition of the mediation effect indicated that the total effect of ATCU on IIPI was 0.756, of which the direct effect was 0.220 (accounting for 29.10%), and the indirect effect through IESR was 0.536 (accounting for 70.90%, 95% CI [0.509, 0.563]). This indicates that IESR played a significant partial mediating role in the process by which ATCU influences IIPI, with ATCU primarily promoting the development of IIPI through enhancing IESR.

**Table 3:** Mediation effect test results of IESR.

Effect type	Path	Effect value	SE	*t*	*p*	95% CI	Proportion
Total effect	ATCU → IIPI	0.756	0.013	60.318	< 0.001	[0.731, 0.781]	100%
Direct effect	ATCU → IIPI	0.22	0.016	13.749	< 0.001	[0.189, 0.252]	29.10%
Indirect effect	ATCU → IESR → IIPI	0.536	0.014	–	–	[0.509, 0.563]	70.90%

*N* = 2,466; SE, standard error; indirect effect calculated using bootstrap method (5,000 repeated samples) for 95% confidence interval; ATCU, Abstract Thinking and Conceptual Understanding; IESR, Inquiry Execution and Strategic Regulation; IIPI, Interdisciplinary Integration and Practical Innovation.

## 6 Discussion

The present study examined the psychological processes through which Abstract Thinking and Conceptual Understanding (ATCU) is associated with Interdisciplinary Integration and Practical Innovation (IIPI) among 2,466 Chinese high school students, with particular attention to the mediating role of Inquiry Execution and Strategic Regulation (IESR). Drawing on self-regulated learning theory (Zimmerman, 2002), knowledge construction theory (Bereiter and Scardamalia, 2014), and learning transfer theory (Barnett and Ceci, 2002), the findings suggest an interpretable pathway linking conceptual understanding with innovative performance through inquiry and regulatory processes. In the following sections, the main findings are discussed in relation to existing literature, followed by the study's theoretical implications, practical relevance, and limitations.

### 6.1 The direct effect of ATCU on IIPI and the knowledge-action gap

Consistent with Hypothesis 1, ATCU showed a significant positive direct association with IIPI, suggesting that students with stronger abstract thinking and deeper conceptual understanding may be better positioned to integrate knowledge across disciplines and generate practically oriented innovative solutions. This finding is broadly consistent with the view that well-structured conceptual schemas provide an important cognitive basis for far transfer (Barnett and Ceci, 2002) and creative problem solving (Honey et al., 2014). When learners internalize abstract principles rather than rely primarily on isolated factual knowledge, they may be more capable of identifying structural commonalities across superficially different problem contexts, a capacity that appears particularly relevant for interdisciplinary innovation in STEM education (Kelley and Knowles, 2016).

At the same time, the direct effect was relatively modest compared with the total effect, suggesting that conceptual understanding alone may not fully explain students' innovative performance. This pattern is in line with concerns raised in prior research regarding a persistent knowledge-action gap (OECD, 2023), and it lends support to the view that abstract knowledge is more likely to contribute to innovation when it is enacted through inquiry and regulatory processes (Bereiter and Scardamalia, 2014). In this sense, the present findings extend research that has primarily emphasized the direct contribution of disciplinary knowledge to academic achievement (Mullis et al., 2020), by indicating that in the context of interdisciplinary innovation, the role of abstract understanding may be partly realized through subsequent self-regulatory engagement.

### 6.2 The mediating role of IESR and the “understanding, inquiry, and reflection” cycle

One notable finding of this study is that IESR significantly mediated the association between ATCU and IIPI and accounted for a substantial proportion of the total effect. This result is consistent with the proposed “understanding, inquiry, and reflection” cycle and offers empirical support for the idea that metacognitive and regulatory processes may play an important intermediary role in translating higher-order cognitive resources into innovative outcomes (Schukajlow et al., 2023).

From a theoretical perspective, this finding suggests that the relationship between knowledge and innovation may be better understood as a dynamic and recursive process than as a simple linear progression from cognitive input to performance output. Abstract conceptual resources appear to provide an important foundation for innovation, but their practical expression may depend on learners' capacity to engage in inquiry execution, such as formulating questions, designing investigations, and monitoring progress, together with strategic regulation, such as evaluating, adjusting, and reflecting on their approaches. This interpretation is consistent with Zimmerman's (2002) cyclical model of self-regulated learning, in which forethought, performance, and self-reflection iteratively shape learners' engagement with complex tasks. It also aligns with Bereiter and Scardamalia's (2014) account of knowledge building, which emphasizes sustained inquiry and the iterative improvement of ideas as central to meaningful knowledge creation.

This mediation pattern also speaks to a broader tendency in interdisciplinary innovation research to emphasize instructional design and curricular arrangements (Kelley and Knowles, 2016; Kwon and Lee, 2025), while giving comparatively less attention to the internal psychological processes through which students mobilize what they know. By specifying how abstract concepts, inquiry execution, and strategic regulation may operate together at the learner level, the present study complements existing instructional perspectives and offers a more process-oriented account of innovation development.

### 6.3 Developmental significance for adolescent learners and theoretical contributions

The findings may be particularly meaningful in the context of high school education, a developmental period characterized by the continued maturation of higher-order thinking and increasingly consequential academic and career-related decision making (Inhelder and Piaget, 1958; Kuhn, 2009; Poll and Petru, 2023). The relative robustness of the mediation model across 2,466 participants suggests that the “understanding, inquiry, and reflection” cycle may represent a plausible psychological process during adolescence. This stage appears to present both challenges and opportunities. On the one hand, adolescents may accumulate considerable disciplinary knowledge without necessarily developing the inquiry and regulatory competencies needed to apply that knowledge in novel interdisciplinary situations (Kim and Joo, 2023). On the other hand, prior work has suggested that metacognitive and regulatory skills remain malleable during adolescence (Poll and Petru, 2023), indicating that interventions targeting IESR may be especially worthwhile during this stage. This possibility is also relevant to broader international efforts to cultivate innovation-related competencies among young people (UNESCO, 2024; World Economic Forum, 2020).

Several theoretical implications emerge from the present findings. First, by examining IESR as a mediating process rather than merely as a parallel predictor, the study offers a more differentiated account of how regulatory processes may connect conceptual understanding with innovative performance in interdisciplinary contexts. Second, the integration of self-regulated learning, knowledge construction, and learning transfer theories within a single empirical framework helps clarify how these perspectives may jointly illuminate the development of innovation-related competence. Third, the articulation of an “understanding, inquiry, and reflection” cycle provides a useful conceptual language for describing how declarative knowledge may be transformed into procedural engagement and, in some cases, into innovative practice.

### 6.4 Practical implications for curriculum, instruction, and assessment

The findings also have practical implications relevant to SDG 4 on Quality Education and SDG 9 on Industry, Innovation, and Infrastructure (UNESCO, 2024). With respect to curriculum design, the results suggest that competency-based STEM curricula may benefit from positioning inquiry and reflection as central components of learning rather than as supplementary extensions to conceptual instruction. Project-based and problem-based learning approaches (Bybee, 2013; English, 2016; Hwang et al., 2024) appear well aligned with this goal, particularly when they include explicit scaffolds for the regulatory processes reflected in IESR.

In classroom instruction, teachers may consider incorporating metacognitive prompts, structured reflection routines, and progressively complex inquiry tasks that encourage students to plan, monitor, and evaluate their approaches to interdisciplinary problems. Such practices may be especially useful for supporting the application of conceptual understanding in authentic contexts. In terms of assessment, the findings point to the value of moving beyond measures that focus primarily on isolated content recall toward forms of assessment that are more sensitive to inquiry processes, regulatory strategies, and innovative performance in context. This direction is broadly consistent with international discussions about rethinking secondary education assessment in response to recent declines in PISA performance (OECD, 2023) and the growing emphasis on competency-oriented education (National Academies of Sciences, 2024).

### 6.5 Limitations and directions for future research

Several limitations should be considered when interpreting the findings. First, the cross-sectional design does not permit strong causal inferences regarding the directionality of the ATCU–IESR–IIPI pathway. Longitudinal studies and intervention-based designs would be valuable for examining the temporal ordering and developmental dynamics of these relationships more directly. Second, although the sample size was substantial, the participants were drawn from Chinese high school students, which may limit the generalizability of the findings to other cultural and educational settings. Cross-cultural comparative research would therefore be helpful in testing the broader applicability of the proposed model. Third, all variables were assessed using self-report measures, which may increase the risk of common method bias. Future studies could strengthen the evidence base by incorporating behavioral indicators, performance-based assessments, and teacher evaluations. Fourth, the present model focuses primarily on cognitive and regulatory processes and does not include motivational, emotional, or social factors that may also be associated with interdisciplinary innovation (Schukajlow et al., 2023). Future research may therefore benefit from considering constructs such as creative self-efficacy, intrinsic motivation, and collaborative inquiry in order to develop a more comprehensive account of innovation-related development. Finally, although IESR was modeled here as a unified mediating construct, inquiry execution and strategic regulation may function somewhat differently across task types and disciplinary contexts. More fine-grained analyses could help clarify these potential variations.

## 7 Conclusion

This study examined the psychological pathway linking Abstract Thinking and Conceptual Understanding (ATCU), Inquiry Execution and Strategic Regulation (IESR), and Interdisciplinary Integration and Practical Innovation (IIPI) among 2,466 Chinese high school students. Drawing on theories of self-regulated learning, knowledge construction, and learning transfer, the findings suggest that ATCU is positively associated with IIPI and that this relationship is substantially mediated by IESR. This mediation pattern is consistent with an “understanding, inquiry, and reflection” cycle, indicating that conceptual understanding may contribute to innovative performance in part through learners' capacity to plan, monitor, adjust, and reflect on their inquiry processes.

From a theoretical perspective, the study contributes to the literature by extending metacognitive research into the domain of interdisciplinary innovation. By conceptualizing IESR as an intervening process rather than merely as a parallel predictor, the study offers a more differentiated account of how regulatory mechanisms may connect conceptual resources with innovative outcomes. From a practical perspective, the findings suggest that secondary education may benefit from moving beyond a predominant focus on the accumulation of disciplinary knowledge and placing greater emphasis on inquiry-based and reflection-oriented learning. Such an orientation is broadly consistent with competency-based STEM education and with the aims of SDG 4 and SDG 9 (UNESCO, 2024).

Several limitations should also be noted. The cross-sectional design limits strong causal interpretation, the single-country sample may constrain the generalizability of the findings, and the reliance on self-report measures raises the possibility of common method bias. Future research could build on the present study by employing longitudinal, intervention-based, and cross-cultural designs, while also incorporating motivational and social dimensions to provide a more comprehensive account of the developmental processes involved. Overall, the study offers empirical support for a process-oriented account of how adolescents' conceptual understanding may be linked to interdisciplinary innovation, and it contributes to ongoing efforts to better understand the development of innovation-related competencies in secondary education.

## Data Availability

The raw data supporting the conclusions of this article will be made available by the authors, without undue reservation.

## References

[B1] Adey P. Shayer M. (1994). Really Raising Standards: Cognitive Intervention and Academic Achievement. London: Routledge.

[B2] Amabile T. M. (1996). Creativity in Context: Update to the Social Psychology of Creativity. Boulder, CO: Westview Press.

[B3] Anderson L. W. Krathwohl D. R. Airasian P. W. Cruikshank K. A. Mayer R. E. Pintrich P. R. . (2001). A Taxonomy for Learning, Teaching, and Assessing: A Revision of Bloom's Taxonomy of Educational Objectives. New York, NY: Longman.

[B4] Azevedo R. (2018). “Using hypermedia as a metacognitive tool for enhancing student learning? The role of self-regulated learning,” in Computers as *M*etacognitive *T*ools for *E*nhancing *L*earning (London: Routledge), 199–209. doi: 10.1207/s15326985ep4004_2

[B5] Barnett S. M. Ceci S. J. (2002). When and where do we apply what we learn? A taxonomy for far transfer. Psychol. Bull. 128, 612–637. doi: 10.1037/0033-2909.128.4.612 12081085

[B6] Bereiter C. Scardamalia M. (2014). “Knowledge building and knowledge creation: one concept, two hills to climb,” in Knowledge *C*reation in *E*ducation, eds. S. C. Tan, H. J. So, and J. Yeo (Singapore: Springer) 35–52. doi: 10.1007/978-981-287-047-6_3

[B7] Beymer P. N. Flake J. K. Schmidt J. A. (2023). Disentangling students' anticipated and experienced costs: the case for understanding both. J. Educ. Psychol. 115:624. doi: 10.1037/edu0000789

[B8] Boix Mansilla V. (2005). Assessing student work at disciplinary crossroads. Change Mag. High. Learn. 37, 14–21. doi: 10.3200/CHNG.37.1.14-21

[B9] Bybee R. W. (2013). The Case for STEM Education: Challenges and opportunities. Arlington, VA: NSTA Press.

[B10] Darbellay F. (2015). The gift of interdisciplinarity: towards an ability to think across disciplines. Int. J. Talent Dev. Creat. 3, 201–211.

[B11] Darbellay F. (2022). The gift of interdisciplinarity: towards an ability to think across disciplines. Int. J. Innov. Stud. 6, 91–99.

[B12] Dignath C. Büttner G. (2008). Components of fostering self-regulated learning among students. A meta-analysis on intervention studies at primary and secondary school level. Metacogn. Learn. 3, 231–264. doi: 10.1007/s11409-008-9029-x

[B13] Dunlosky J. Rawson K. A. Marsh E. J. (2024). Improving students' learning with effective learning techniques: promising directions from cognitive and educational psychology. Psychol. Sci. Public Interest 25, 3–48. 10.1177/1529100612453266 26173288

[B14] English L. D. (2016). STEM education K-12: perspectives on integration. Int. J. STEM Educ. 3:3. doi: 10.1186/s40594-016-0036-1

[B15] Flavell J. H. (1979). Metacognition and cognitive monitoring: a new area of cognitive-developmental inquiry. Am. Psychol. 34, 906–911. doi: 10.1037/0003-066X.34.10.906

[B16] Hayes A. F. (2018). Introduction to Mediation, Moderation, and Conditional Process Analysis: A Regression-based Approach, 2nd Edn. New York, NY: The Guilford Press.

[B17] Honey M. Pearson G. Schweingruber H. eds. (2014). STEM Integration in K-12 Education: Status, Prospects, and an Agenda for Research. Washington, DC: National Academies Press.

[B18] Hwang J. Choo S. Morano S. Liang M. Kabel M. (2024). From silos to synergy in STEM education: promoting interdisciplinary STEM education to enhance the science achievement of students with learning disabilities. Remedial Spec. Educ. 46, 187–199. doi: 10.1177/09388982241245452

[B19] Inhelder B. Piaget J. (1958). The Growth of Logical Thinking from Childhood to Adolescence. New York, NY: Basic Books. doi: 10.1037/10034-000

[B20] Kelley T. R. Knowles J. G. (2016). A conceptual framework for integrated STEM education. Int. J. STEM Educ. 3:11. doi: 10.1186/s40594-016-0046-z

[B21] Kim H. J. Joo Y. H. (2023). Near and far transfer in South Korean teacher education: the moderated mediation effects of social capital and intensive reflection. SAGE Open 13, 1–14. doi: 10.1177/21582440231188916

[B22] Klein J. T. (2005). Humanities, Culture, and Interdisciplinarity: The Changing American Academy. Albany, NY: State University of New York Press. doi: 10.1353/book4993

[B23] Kline R. B. (2016). Principles and Practice of Structural Equation Modeling, 4th Edn. New York, NY: The Guilford Press.

[B24] Krawczyk D. C. (2018). Reasoning: The Neuroscience of How We Think. London: Academic Press. doi: 10.1016/C2015-0-05936-6

[B25] Kuhn D. (2009). “Adolescent thinking,” in Handbook of Adolescent Psychology, 3rd Edn, eds. R. M. Lerner and L. Steinberg (Hoboken, NJ: John Wiley and Sons), 152–186. doi: 10.1002/9780470479193.adlpsy001007

[B26] Kwon H. Lee Y. (2025). A meta-analysis of STEM project-based learning on creativity. STEM Educ. 5, 275–290. doi: 10.3934/steme.2025014

[B27] Marginson S. Tytler R. Freeman B. Roberts K. (2013). STEM: Country comparisons. Melbourne: Australian Council of Learned Academies.

[B28] Mullis I. V. S. Martin M. O. Foy P. Kelly D. L. Fishbein B. (2020). TIMSS 2019 International Results in Mathematics and Science. Chestnut Hill, MA: TIMSS and PIRLS International Study Center, Boston College. Available online at: https://timssandpirls.bc.edu/timss2019/international-results/ (Accessed November 2, 2025).

[B29] National Academies of Sciences Engineering, and Medicine. (2024). Equity in K-12 STEM Education: Framing Decisions for the Future. Washington, DC: The National Academies Press.

[B30] Nersessian N. J. Patton C. (2023). Model-based reasoning in interdisciplinary engineering sciences. Top. Cogn. Sci. 15, 124–147. doi: 10.1016/B978-0-444-51667-1.50031-8

[B31] OECD (2018). The Future of Education and Skills: Education 2030. Paris: OECDPublishing.

[B32] OECD (2019). PISA 2018 Results (Volume I): What Students Know and Can Do. Paris: OECDPublishing.

[B33] OECD (2023). PISA 2022 Results (Volume I): The State of Learning and Equity in Education. Paris: OECDPublishing.

[B34] Panadero E. (2017). A review of self-regulated learning: six models and four directions for research. Front. Psychol. 8:422. doi: 10.3389/fpsyg.2017.00422 28503157 PMC5408091

[B35] Phung T. Choi H. Wu M. Singla A. Brooks C. (2025). “Plan more, debug less: applying metacognitive theory to AI-assisted programming education,” in International Conference on Artificial Intelligence in Education (Cham: Springer Nature Switzerland), 3–17. doi: 10.1007/978-3-031-98414-3_1

[B36] Poll G. H. Petru J. (2023). Assessing adolescent metacognitive skills to support transition planning: age-related change and domain specificity. Career Dev. Transit. Except. Individ. 46, 102–113. doi: 10.1177/15257401221120368

[B37] Repko A. F. Szostak R. (2020). Interdisciplinary Research: Process and Theory, 4th Edn. Thousand Oaks, CA: SAGE Publications.

[B38] Schraw G. Dennison R. S. (1994). Assessing metacognitive awareness. Contemp. Educ. Psychol. 19, 460–475. doi: 10.1006/ceps.1994.1033

[B39] Schukajlow S. Rakoczy K. Pekrun R. (2023). Emotions and motivation in mathematics education: where we are today and where we need to go. ZDM Math. Educ. 55, 249–267. doi: 10.1007/s11858-022-01463-2 PMC9845103 36684477

[B40] Spelt E. J. H. Biemans H. J. A. Tobi H. Luning P. A. Mulder M. (2009). Teaching and learning in interdisciplinary higher education: a systematic review. Educ. Psychol. Rev. 21, 365–378. doi: 10.1007/s10648-009-9113-z

[B41] Swiecki Z. Khosravi H. Chen G. Martinez-Maldonado R. Lodge J. M. Milligan S. . (2022). Assessment in the age of artificial intelligence. Comput. Educ. Artif. Intell. 3:100075. doi: 10.1016/j.caeai.2022.100075

[B42] Torrance E. P. (1974). Torrance Tests of Creative Thinking: Norms-Technical Manual. Lexington, MA: Personnel Press.

[B43] UNESCO. (2024). Transforming Education Towards SDG 4: Highlights. Paris: UNESCO Publishing.

[B44] Van der Stel M. Veenman M. V. J. (2010). Development of metacognitive skillfulness: a longitudinal study. Learn. Individ. Differ. 20, 220–224. doi: 10.1016/j.lindif.2009.11.005

[B45] van der Stel M. Veenman M. V. J. (2014). Metacognitive skills and intellectual ability of young adolescents: a longitudinal study from a developmental perspective. Eur. J. Psychol. Educ. 29, 117–137. doi: 10.1007/s10212-013-0190-5

[B46] Watson G. Glaser E. M. (1980). Watson-Glaser Critical Thinking Appraisal. San Antonio, TX: Psychological Corporation.

[B47] Wiedbusch M. D. Dever D. Delgado T. Azevedo R. (2025). Modeling the dynamics and autoregressive tendencies of metacognitive judgment accuracy during complex learning. Int. J. Artif. Intell. Educ. 35, 1–36. doi: 10.1007/s40593-025-00522-5

[B48] World Economic Forum (2020). The Future of Jobs Report 2020. Geneva: World Economic Forum.

[B49] Yakman G. (2007). “STEAM education: an overview of creating a model of integrative education,” in Pupils' Attitudes towards Technology (PATT) Conference (Salt Lake City, UT), 1–28.

[B50] Zimmerman B. J. (2000). “Attaining self-regulation: a social cognitive perspective,” in Handbook of self-regulation, eds. M. Boekaerts, P. R. Pintrich, and M. Zeidner (San Diego, CA: Academic Press) 13–39. doi: 10.1016/B978-012109890-2/50031-7

[B51] Zimmerman B. J. (2002). Becoming a self-regulated learner: an overview. Theory Pract. 41, 64–70. doi: 10.1207/s15430421tip4102_2

