# Correction: From conceptual understanding to interdisciplinary innovation: the mediating mechanism of IESR among high school students

**DOI:** 10.3389/fpsyg.2026.1888522

**Published:** 2026-06-11

**Authors:** Jiyuan Xu, Yurui Mou, Lei Yuan

**Affiliations:** 1 Faculty of Education, Guangxi Normal University, Guilin, China; 2 Research and Development Center for Educational Informatization, Guangxi Normal University, Guilin, China; 3 Ziyang College of Dental Technology, Ziyang, China; 4 Key Laboratory of Educational Blockchain and Intelligent Technology, Ministry of Education, Guangxi Normal University, Guilin, China

**Keywords:** abstract thinking, conceptual understanding, interdisciplinary integration, mediating effect, practical innovation, scientific inquiry, strategic regulation

Affiliation “Ziyang College of Dental Technology, Ziyang, China” was omitted for author Yurui Mou. This affiliation has now been added for author Yurui Mou.

Author Yurui Mou was erroneously assigned to affiliation “Key Laboratory of Educational Blockchain and Intelligent Technology, Ministry of Education, Guangxi Normal University, Guilin, China”. This affiliation has now been removed for author Yurui Mou.

The original version of this article has been updated.

